# Anti-Inflammatory Effects of 4-Methylcyclopentadecanone on Edema Models in Mice

**DOI:** 10.3390/ijms141223980

**Published:** 2013-12-09

**Authors:** Yukui Ma, Yue Li, Xiufeng Li, Yingliang Wu

**Affiliations:** 1 Department of Pharmacology, Shenyang Pharmaceutical University, Wenhua Road 103, Shenyang 110016, China; E-Mail: yukuima@sina.com; 2 Shandong Provincial Key Laboratory of Chemical Drug, Shandong Institute of Pharmaceutical Industry, Xinluo Road 989, Jinan 250101, China; 3 Department of Pharmacy, Zhangqiu People’s Hospital, Huiquan Road 1920, Zhangqiu 250200, China; E-Mail: cbandly@163.com; 4 Shandong Hongjitang Pharmaceutical Group Co., Ltd., Hualong Road 360, Jinan 250100, China; E-Mail: lang9510@sina.com

**Keywords:** 4-methylcyclopentadecanone, inflammation, paw edema, ear edema, toxicity

## Abstract

The present study evaluated the anti-inflammatory effects of 4-methylcyclopentadecanone (4-MCPC) on edema models in mice and aimed to determine the safety of 4-MCPC after acute exposure. The acute toxicity of 4-MCPC was evaluated by oral administration to rats of single doses of 0, 5, 50, 500 and 5000 mg/kg. Toxic symptoms were observed for 14 days. The anti-inflammatory activity was evaluated in xylene-induced mouse ear edema and carrageenan-induced mouse paw edema. The animals were treated with 4-MCPC once every day for seven consecutive days. Edema index, % inhibition, IL-1β, TNF-α, PGE_2_ and MPO levels in paws were detected after the treatment with xylene or carrageenan. Our results indicated that the LD_50_ value of 4-MCPC in rats is greater than 5000 mg/kg. The ED_50_ of 4-MCPC in xylene-induced mouse ear edema model was 7.5 mg/kg. 4-MCPC (8 or 16 mg/kg) remarkably inhibited carrageenan-induced mouse paw edema. Further study revealed that 4-MCPC treatment also decreased IL-1β, TNF-α, PGE_2_ and MPO levels in mice paws. Intragastric administration of 4-MCPC exhibited more significant anti-inflammatory activity than muscone at a dose of 16 mg/kg. Taken together, our results suggest that 4-MCPC has potent anti-inflammatory activity and the mechanisms might be related to the decreases of the levels of IL-1β, TNF-α, PGE_2_ and MPO in inflamed paws.

## 1. Introduction

Inflammation is the first response of the immunological defense system to microbial infections, burns, allergens, mechanical injuries and other noxious stimuli [1]. Inflammation is involved in the pathogenesis of many diseases, such as diabetes, cardiovascular, neurodegenerative, cancer and other life-threatening diseases [2]. Inflammation is a complex series of cascade reactions, including enzyme activation, release of chemical mediators, effusion of fluids, cell migration, and tissue damage and repair [3]. During the inflammatory process, macrophages play a crucial role. Macrophages activated by stimuli produce inflammatory mediators such as nitric oxide (NO) and prostaglandin E_2_ (PGE_2_), and various cytokines such as interleukin-1β (IL-1β), interleukin-6 (IL-6), interleukin-10 (IL-10) and tumor necrosis factor-α (TNF-α) [4,5]. Non-steroidal anti-inflammatory drugs (NSAIDs) are the main available potent synthetic drugs in the treatment of inflammatory diseases. However, the use of NSAIDs as anti-inflammatory agents has not been successful in their clinical use because of the serious adverse side effects such as gastric lesions and reappearance of symptoms after discontinuation [6]. Therefore, there is a worldwide search for new anti-inflammatory drugs as an alternative to NSAIDs.

Musk is one of the rare medicinal herbs, and it has been widely used for treating fractures, sprains, angina pectoris and myocardial infarction for thousands of years, especially in China [7,8]. Muscone, whose chemical structure is recognized as 3-methylcyclopentadecanone, is believed to be the main active ingredient of musk [7]. Muscone is reported to be a potent anti-inflammatory agent that reduces the levels of anti-inflammatory cytokines *in vitro* and *in vivo* [9]. 4-Methylcyclopentadecanone (4-MCPC) is the isomer of muscone and a by-product of muscone synthesis that was discarded in the past. Experimental studies have shown that 4-MCPC has similar pharmacological effects as muscone in some aspects. A patent application was filed in China for the use of 4-MCPC for the treatment of ischemic cerebrovascular disease, rheumatic arthritis, tumor and Alzheimer’s disease [10]. Our previous study also proved that 4-MCPC has similar effects and mechanisms with muscone in an ischemia/reperfusion (I/R) injury model. However, the anti-inflammatory effect and the associated mechanisms of 4-MCPC compared with muscone have not been explored. This is the first report of the anti-inflammatory effects and the associated mechanisms of 4-MCPC in animal edema models.

## 2. Results

### 2.1. Acute Toxicity

4-MCPC at doses of 5–5000 mg/kg, *p.o.*, given to rats showed no toxic symptoms during the monitoring period of 14 days after administration. The LD_50_ value of 4-MCPC in rats was estimated at >5 g/kg, *p.o.*, which was 667-fold more than the ED_50_ of xylene-induced mouse ear edema model.

### 2.2. Effects on Xylene-Induced Mouse Ear Edema

**Figure 1.**
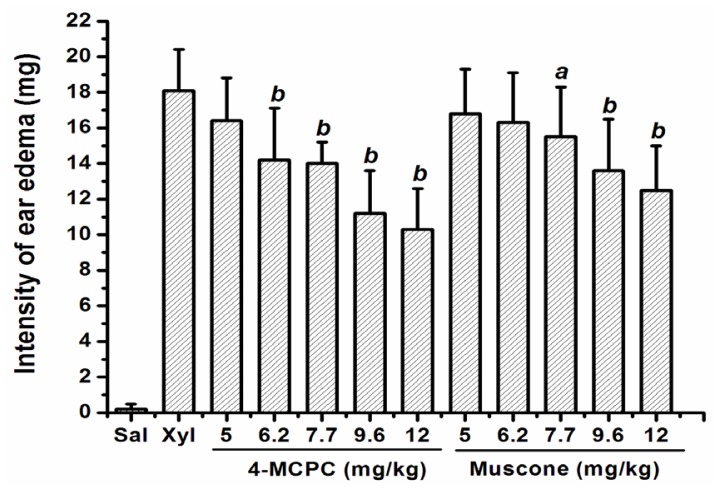
Effects of 4-MCPC and muscone on xylene-induced mouse ear edema. Administration of 4-MCPC (12.0, 9.6, 7.7 and 6.2 mg/kg) and muscone (12.0, 9.6 and 7.7 mg/kg), respectively, reduced ear edema. Bars represent mean ± SD for each group, *n* = 10. *^a^p* < 0.05; *^b^p* < 0.01 *versus* xylene model group. Sal: saline; Xyl: xylene.

As shown in Figure 1, intragastric administration of 4-MCPC (12.0, 9.6, 7.7 and 6.2 mg/kg) and muscone (12.0, 9.6 and 7.7 mg/kg), respectively, reduced ear edema (*p* < 0.05 or *p* < 0.01). The ED_50_ of 4-MCPC and muscone were 7.5 mg/kg and 11.5 mg/kg, respectively.

### 2.3. Effects on Carrageenan-Induced Mouse Paw Edema

**Figure 2.**
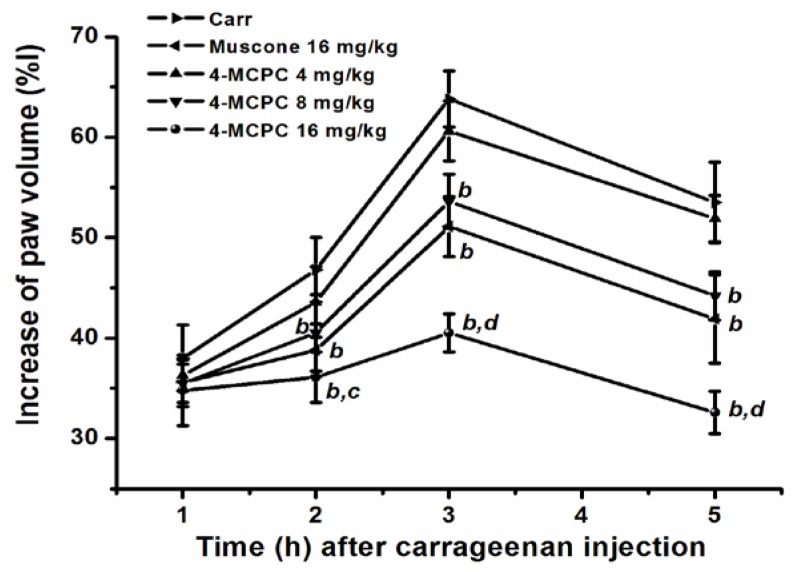
Effects of 4-MCPC on carrageenan-induced mouse paw edema. Treatment with 4-MCPC (8 and 16 mg/kg) and muscone (16 mg/kg), respectively, reduced paw edema at 2, 3 or 5 h after carrageenan injection. For each group, *n* = 10. *^b^p* < 0.01 *versus* carrageenan model group; *^c^p* < 0.05; *^d^p* < 0.01, *versus* muscone group. Carr: carrageenan.

The effects on carrageenan-induced mouse paw edema are shown in Figure 2. Compared with the model group, the intragastric administration of 4-MCPC (8 and 16 mg/kg) and muscone (16 mg/kg), respectively, reduced paw edema at 2, 3 or 5 h after carrageenan injection (*p* < 0.01). Intragastric administration of 4-MCPC exhibited more significant anti-inflammatory activity than muscone at a dose of 16 mg/kg (*p* < 0.05 or *p* < 0.01).

### 2.4. Effects on Myeloperoxidase (MPO) Activity in Carrageenan-Induced Mouse Paws

**Figure 3.**
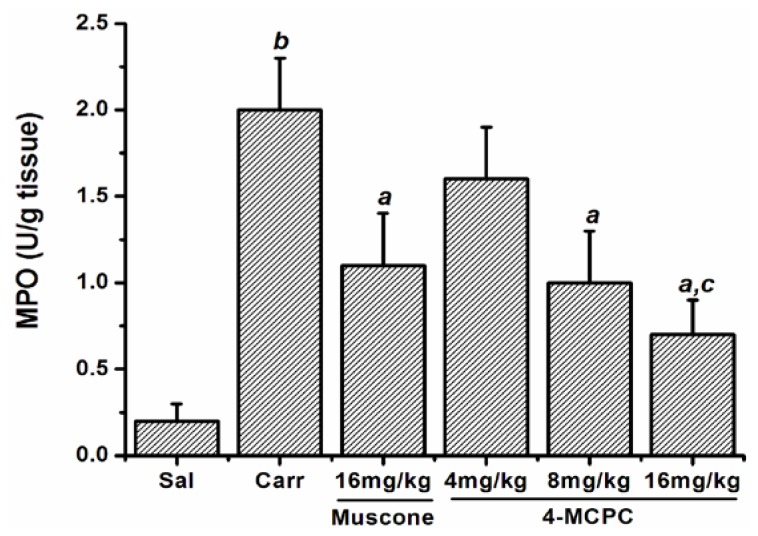
Effects of 4-MCPC on MPO activity in carrageenan-induced mouse paws. MPO activity was evaluated at 5 h after injection of carrageenan. MPO activity was reduced by 4-MCPC (8 and 16 mg/kg) and muscone (16 mg/kg). Bars represent mean ± SD for samples from six paws in each group. *^a^p* < 0.01 *versus* carrageenan group; *^b^p* < 0.01 *versus* saline control group; *^c^p* < 0.05 *versus* muscone group. Sal: saline; Carr: carrageenan.

As shown in Figure 3, compared with the control group, injection of carrageenan enhanced the MPO activity in the paws. The MPO activity was reduced by 4-MCPC at 8 and 16 mg/kg (*p* < 0.01). Intragastric administration of muscone at 16 mg/kg also decreased MPO activity (*p* < 0.01). The intragastric treatment of animals with 4-MCPC exhibited more effects of MPO activity than with muscone at 16 mg/kg (*p* < 0.05).

### 2.5. Effects on IL-1β, TNF-α and PGE_2_ Levels in Carrageenan-Induced Mouse Paws

**Figure 4.**
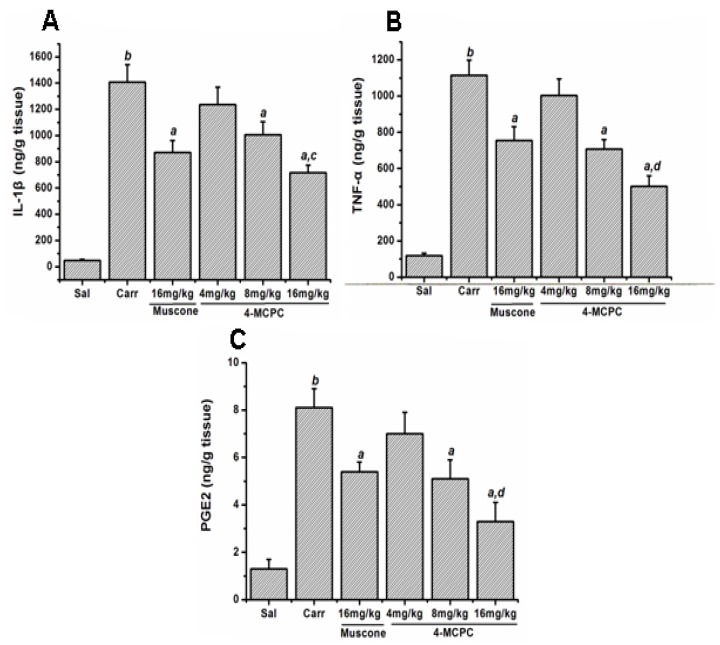
Effects of 4-MCPC on IL-1β (**A**); TNF-α (**B**); and PGE_2_ (**C**) levels in carrageenan-induced mouse paws. Treatment with 4-MCPC (8 and 16 mg/kg) and muscone (16 mg/kg), respectively, reduced IL-1β, TNF-α and PGE_2_ levels in edema paws 5 h after carrageenan injection. For each group, *n* = 6. *^a^p* < 0.01 *versus* carrageenan model group; *^b^p* < 0.01 *versus* saline control group; *^c^p* < 0.05; *^d^p* < 0.01 *versus* muscone group. Sal: saline; Carr: carrageenan.

As shown in Figure 4, injection of carrageenan increased the IL-1β, TNF-α and PGE_2_ levels in the paws, when compared to control group (*p* < 0.01). Compared with the model group, intragastric administration of 4-MCPC (8 and 16 mg/kg) and muscone (16 mg/kg), respectively, reduced IL-1β, TNF-α and PGE_2_ levels in the paws (*p* < 0.01). There was significant difference in IL-1β, TNF-α and PGE_2_ levels between the groups of 4-MCPC and muscone at a dose of 16 mg/kg (*p* < 0.05 or *p* < 0.01).

### 2.6. Histopathology Analysis

**Figure 5.**
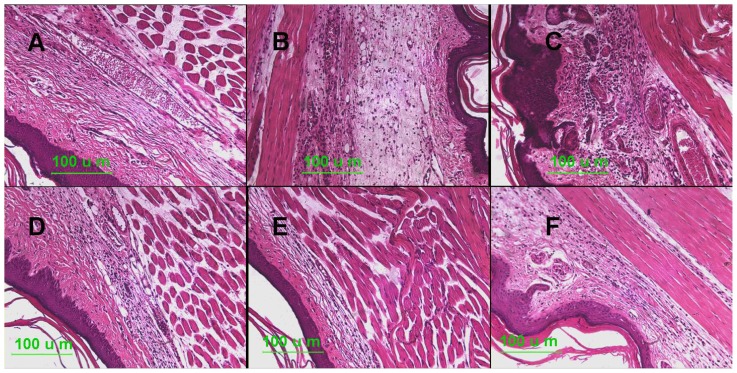
Histological changes in edema paws 5 h after injection of carrageenan. Paws were harvested 5 h after injection of carrageenan and subjected to histochemical staining of paw tissues. (**A**) Saline control group; (**B**) Carrageenan model group; (**C**) 4 mg/kg of 4-MCPC-treated group; (**D**) 8 mg/kg of 4-MCPC-treated group; (**E**) 16 mg/kg of 4-MCPC-treated group; and (**F**) 16 mg/kg of muscone-treated group; magnification ×20.

The histopathological results of edema paws are shown in Figure 5. According to Figure 5A, no cellular infiltration and edema were observed in the control group. In contrast, polymorphonuclear (PMN) infiltration and swelling followed the carrageenan injection (Figure 5B). After treatment with 4-MCPC at the doses of 8 and 16 mg/kg, the edema and PMN infiltration was significantly reduced (Figure 5D,E). However, slight improvements in edema and PMN infiltration were observed in the 4-MCPC-treated group (4 mg/kg) (Figure 5C). The reference drug muscone at a dose of 16 mg/kg exhibited the same effect with 4-MCPC-treated group (8 mg/kg) (Figure 5F).

## 3. Discussion

In the present study, anti-inflammatory effects and the underlying mechanisms of 4-MCPC were investigated on xylene-induced mouse ear edema and carrageenan-induced mouse paw edema for the first time. The acute oral toxicity was also evaluated in this study. Our data reveal that the LD_50_ value of 4-MCPC is greater than 5000 mg/kg and 667-fold more than the ED_50_ of xylene-induced mouse ear edema model. It can be classified as a safe chemical with low toxicity according to OECD (2008). Our data also demonstrate that 4-MCPC inhibits inflammation by decreasing the IL-1β, TNF-α, PGE_2_ and MPO levels in mouse paw tissues. In addition, our studies reveal that intragastric administration of 4-MCPC exhibits more significant anti-inflammatory activity than muscone at the same dose level.

Myeloperoxidase is a specific marker of myeloid cells. It is abundant in azurophilic granules of monocytes and neutrophils after the activation by stimuli; therefore, tissue MPO level has been used as an inflammatory marker [11]. Our study demonstrated that the injection of carrageenan enhanced the MPO activity in the paws, and treatment with 4-MCPC at 8 and 4 mg/kg showed a significant decrease in MPO activity compared with the model group. The result suggested that 4-MCPC could act by inhibiting the neutrophil infiltration into the inflammatory site, which was in accordance with the results of histological examination (Figure 5).

The inflammation response is phylogenetically and ontogenetically a principle defense mechanism that is controlled by inflammatory mediators such as NO and PGE_2_, and various cytokines such as IL-1β, IL-6, TNF-α [12]. Therefore, inflammatory mediators and cytokines play important roles in the inflammatory response. Previous studies had shown that muscone was a potent anti-inflammatory agent that reversed IL-1β-induced upregulation of IL-1β and TNF-α *in vitro* and downregulated the expression of PGE_2_, IL-1β, TNF-α *in vivo* and downregulated the expression of PGE_2_, IL-1β, TNF-α *in vivo* [9]. 4-MCPC is the isomer of muscone and has similar pharmacological effects as muscone. We therefore hypothesized that 4-MCPC exhibited potent anti-inflammatory activity and the possible mechanisms might be related to the decrease of the levels of IL-1β, TNF-α, and PGE_2_ in inflamed tissues.

Xylene, a common inflammatory agent, provokes acute inflammatory response in the mouse ear, which leads to serious edematous changes of skin when applied to the surfaces of the ear [13]. The ear edema model induced by xylene has certain advantages in the evaluation of anti-inflammatory steroids as well as non-steroidal anti-inflammatory agents and has good predictive values in the screening of antiphlogistic new drugs [13,14]. The carrageenan-induced mouse paw edema test is the most widely used primary test to evaluate new anti-inflammatory drugs [15]. Carrageenan injection into the mice paw causes an acute and local inflammatory response. The model is highly reproducible and has been well established as a valid model to study pro-inflammatory mediators and cytokine generation in paw tissue in inflammatory conditions [16–18]. In this study, we used the xylene-induced ear edema model for the screening of dose range of 4-MCPC. Furthermore, due to its possible mechanisms related to the effects on mediators and cytokines, we chose a carrageenan-induced mouse paw edema model to evaluate the anti-inflammatory action of 4-MCPC.

The development of carrageenan-induced edema is a biphasic event. The initial phase (0–1 h) is attributed to the release of serotonin, histamine, bradykinin and substance P. The late phase (after 1 h) is mainly due to the neutrophil infiltration into the inflammatory site and the production of large amounts of pro-inflammatory mediators such as PGE_2_ and various cytokines such as IL-1β, IL-6, IL-10 and TNF-α [16,19,20]. TNF-α is produced mainly by mononuclear phagocytes and can cause immune responses by stimulating macrophages and T cells. TNF-α can also induce secretion of other inflammatory cytokines [17]. Nuclear transcription factor-kappa B (NF-κB) and mitogen-activated protein kinase (MAPK) signal pathways are two important signalling pathways involved in inflammation response [21]. NF-κB is an important transcription factor and activated NF-κB up-regulates the expression of proinflammatory cytokine genes, such as IL-1β, IL-6 and TNF-α [22]. The classical MAPKs are comprised with three family members: c-Jun NH_2_-terminal kinase (JNK), mitogen-activated protein kinase (p38-MAPK) and extracellular signal-regulated kinase p42/p44 (ERK1/2) [23]. Phosphorylation of MAPKs can promote the production of pro-inflammatory cytokines [24,25]. IL-1β and TNF-α induce the expression of cyclooxygenase 2 (COX-2), and COX-2 in turn catalyzes the synthesis of PGE_2_[26,27]. Previous studies had shown muscone blocked the generation of pro-inflammatory cytokines by inhibiting JNK and ERK1/2 signal pathways [9]. Our study demonstrated that 4-MCPC inhibited IL-1β, TNF-α and PGE_2_ production in a dose-dependent manner. In addition, 4-MCPC effectively inhibited the increase of paw volume at 2, 3 and 5 h after carrageenan injection (Figure 2). Combined with the results of histological examination, the study fully justified that 4-MCPC showed a significant anti-inflammatory activity at the second phase (after 1 h) of the edema development. We hypothesized the plausible mechanism of 4-MCPC might be related to the suppression of IL-1β and TNF-α production by inhibiting JNK and ERK1/2 signal pathways. However, the precise mechanisms need to be clarified in future studies.

NSAIDs are among the most widely used drugs in the treatment of inflammatory diseases. Their main pharmacological effects come from inhibiting the enzymatic activity of COX. NSAIDs, commonly used as positive control anti-inflammatory agents, showed potent anti-inflammatory effects on xylene-induced ear and carrageenan-induced mouse paw edema model [16,17,28]. Unfortunately, the use of NSAIDs is limited by gastrointestinal adverse effects and about 20% of regular users of NSAIDs will develop duodenum or gastric ulcer [29,30]. NSAIDs, such as diclofenac (100 mg/kg), Aspirin (200 mg/kg) and indomethacin (30 mg/kg, i.p.) also can be used to duplicate acute gastric ulcer model in rats [31–33]. Our study demonstrated that 4-MCPC showed similar effects with NSAIDs on the two models, Furthermore, it is a chemical with low toxicity (LD_50_ > 5000 mg/kg). A 28 days longer-term toxicity pilot studies in rats had been conducted in our laboratory, and the results indicated that there were no obvious toxic effects at the dosage of 800 mg/kg. Above all, the preliminary experiments revealed that 4-MCPC had similar anti-inflammatory effects with NSAIDs and lower toxicity. We plan to carry out overall experiments to assess its safety as a potential anti-inflammatory agent.

## 4. Materials and Methods

### 4.1. Animals

Male Kunming (KM) mice weighing 18–22 g and Sprague-Dawley (SD) rats weighing 250–280 g were obtained from Vital River Laboratory Animal Technology Co., Ltd. (Beijing, China). All animals were housed in groups in a room under a 12:12 h light/dark cycle (temperature 23–25 °C and humidity of 50%–60%). The animals were provided access to standard laboratory rodent chow and water *ad libitum*. The animals were acclimated to the animal facility for at least seven days (rats) or three days (mice) before being used in experiments. All animal-related experimental procedures were approved by the Institutional Animal Care and Use Committee (IACUC) of Shandong Institute of Pharmaceutical Industry.

### 4.2. Drugs

4-MCPC and muscone were provided by Shandong Hongjitang Pharmaceutical Group Co., Ltd. (Jinan, China). All the tested articles were suspended in 1% sodium carboxymethylcellulose suspension in distilled water at the required concentrations, respectively. Carrageenan was purchased from Tokyo Kasei Kogyo Co., Ltd. (Tokyo, Japan). Xylene was obtained from Tianjin Guangcheng Chemical Reagent Co., Ltd. (Tianjin, China). MPO, IL-1β, TNF-α and PGE_2_ kits, purchased from Shanghai Yanjin Biological Technology Co., Ltd. (Shanghai, China), were used for the evaluation of biochemical parameters.

### 4.3. Acute Oral Toxicity Study

Acute oral toxicity study was performed according to OECD 420. Briefly, normal healthy rats were selected and kept in their cages for at least seven days prior to dosing to allow for acclimatization to the laboratory conditions. A pilot study was conducted to determine the dose to be used in the main study. In the pilot study, the effect of each dose was detected in single animal of each sex. The dose level to be used as the starting dose is selected from one of four fixed levels: 5, 50, 500 and 5000 mg/kg. The higher dose was chosen, if the starting dose did not produce severe toxicity and 2000 mg/kg was the dose destination. In this pilot experiment, the dose with evident toxicity but not lethality was identified and used in the main study. In the main study, 10 rats (five females and five males) were used for the dose level determined through the pilot study. The rats were observed carefully for the symptoms of toxicity for up to 14 days.

### 4.4. Xylene-Induced Mouse Ear Edema

The xylene-induced ear edema test was performed as described previously with slight modification [28]. Twelve groups of 10 mice each were administered orally with 1% carboxymethylcellulose sodium solution (20 mL/kg, control and model group), 4-MCPC (12.0, 9.6, 7.7, 6.2 and 5.0 mg/kg) and muscone (12.0, 9.6, 7.7, 6.2 and 5.0 mg/kg), respectively, once every day for seven consecutive days. One hour after the last intragastric administration, 0.03 mL of xylene was applied to the posterior and anterior surfaces of the right ear of each mouse except a control group. The left ear was considered as a control. Thirty minutes after xylene application, the mice were killed, and both ears were removed. Ear disks of 7.0 mm in diameter were cut off and weighed. The weight difference between the right and left ear disks of the same mice was assessed as intensity of edema. Intensity of edema = *V*_c_ − *V*_t_, where *V*_c_ and *V*_t_ represent the weight of the right ear and the left ear, respectively.

### 4.5. Carrageenan-Induced Mouse Paw Edema

Mouse hind paw edema was induced by carrageenan injection, as described previously [20]. Six groups of 10 mice each were administered orally with 1% carboxymethylcellulose sodium solution (20 mL/kg, control and model group), 4-MCPC (4, 8 and 16 mg/kg) and muscone (16 mg/kg), respectively, once every day for seven consecutive days. One hour after the last intragastric administration, 0.05 mL of carrageenan (1% in normal saline) was injected to the plantar side of the right hind paw except control group. Before and 1, 2, 3 or 5 h after carrageenan injection, the paw edema volumes were measured with a plethysmometer (MK-101P, NatureGene Corp., Beijing, China). The inhibition of inflammation was assessed with the increase of paw volumes, which were calculated using the following formula [18,34].

Increase of paw volume (%I) = (*V_t_* − *V*_o_)/*V*_o_ × 100%, where *V_t_* and *V*_o_ represent the final and initial volumes of each mouse, respectively.

### 4.6. Determination of the IL-1β, TNF-α, PGE2 and MPO Levels in Mouse Paw

IL-1β, TNF-α, PGE_2_ and MPO levels in mouse paw tissues were determined as described previously with slight modification [22]. Five hours after carrageenan injection, six mice of each group were sacrificed, and the tissue samples were collected and weighed, snap frozen in liquid nitrogen and stored at −80 °C to be processed for preparation of homogenates. Paw tissues (10% (*w*/*v*)) were homogenized in sodium phosphate buffer (0.1 M PBS, pH = 7.4). The homogenates were centrifuged at 9000× *g* for 20 min at 4 °C. The supernatants were collected, and IL-1β, TNF-α, PGE_2_ and MPO levels were measured by respective ELISA kits according to the kit instructions.

### 4.7. Histopathologic Examination

Histological analysis of edema paws was performed to evaluate the anti-inflammatory effects. Five hours after carrageenan injection, four mice of each group were sacrificed, and the edema paws were fixed in 10% formaldehyde for one week. Then, the tissues were cut into 4-μm thick slices and placed on adhesive glass. Standard HE staining was performed for morphological observation.

### 4.8. Statistical Analysis

All data were analyzed using SPSS 13.0 software, version 13.0 (SPSS Inc., Chicago, IL, USA), and the data were expressed as the mean ± standard deviation (SD). The differences between the treatment and the model groups were analyzed using one-way analysis of variance (ANOVA). The probability of *p* < 0.05 was considered statistically significant.

## 5. Conclusions

In conclusion, these results indicated that 4-MCPC exhibited potent anti-inflammatory activity and the mechanisms might be related to the decrease of the levels of IL-1β, TNF-α, PGE_2_ and MPO in inflamed tissues. Our studies also suggest that 4-MCPC is a safe chemical with low toxicity and may be developed into a pharmacological agent for the treatment of inflammatory diseases.
